# Factors that influence care for second and third trimester termination of pregnancy for medical reasons in Canada: A qualitative investigation

**DOI:** 10.1371/journal.pone.0349167

**Published:** 2026-05-15

**Authors:** Madeleine Ennis, Elise Lavoie-Lebel, Regina Renner, Sarah Munro, Carmelina Barone, Elana Ilott, Jennifer Chisholm, Brigid Dineley, Liv Knutzen, Julie Robertson, Jessica Liauw

**Affiliations:** 1 University of British Columbia, Department of Obstetrics and Gynaecology, Vancouver, British Columbia, Canada; 2 University of Washington, School of Public Health, Seattle, Washington, United States of America; 3 Patient Partners, University of British Columbia, Faculty of Medicine, Vancouver, British Columbia, Canada; 4 Lakehead University, Department of Gender & Women’s Studies, Thunder Bay, Ontario, Canada; Foundation for Research in Health Systems, INDIA

## Abstract

**Background:**

Termination of pregnancy in the 2^nd^/3^rd^ trimester for medical reasons is an essential health service. Our objective was to explore the factors that influence 2^nd^/3^rd^ trimester abortion care for medical reasons in Canada from the perspective of healthcare providers.

**Methods:**

We conducted one-on-one, semi-structured interviews with healthcare providers across the 10 Canadian academic maternal fetal medicine (MFM) centres. The interviews were conducted in English or French, and we collected participant demographic information through an online survey. We used reflexive thematic analysis to identify healthcare providers’ perspectives of barriers and facilitators that impact care for 2^nd^/3^rd^ trimester abortion for medical reasons at their centres. Our analysis was informed by a realist standpoint. We used NVivo14 Software to organize our analysis.

**Findings:**

We recruited 28 participants – 10 MFM specialists, 9 medical geneticists/genetic counsellors, and 9 nurses/social workers. We identified six main themes describing factors that influence termination care: 1) *Provider commitment amid burnout and discomfort* regarding abortion care; 2) *Provider availability and team structure*; 3) *Logistical factors that support coordinated and compassionate care,* including the availability of a dedicated care coordinator, are instrumental to how care is delivered; 4) *Advantages and challenges of local guidelines* highlights a tension between desiring structured equitable guidelines and facilitating flexibility to tailor care to meet individual needs; 5) *Considerations for patient equity* by adjusting care based on patients’ lived experiences; 6) *Communicating with and supporting patients* emphasizes the importance of patient-centered communication and opportunities to help patients navigate their grief and bereavement.

**Conclusions:**

Healthcare providers identified key factors that promote or hinder 2^nd^/3^rd^ trimester abortion care for medical reasons in Canada. Understanding which barriers to address and facilitators to amplify will inform efforts to optimize equitable, patient-centred, supportive, and comprehensive healthcare services.

## Introduction

Termination of pregnancy for medical reasons is an essential and time-sensitive healthcare service. These terminations often occur in the 2^nd^ and 3^rd^ trimester following investigations in routine obstetric care, such as prenatal genetic screening or anatomical ultrasound scans [1]. Patients are then usually referred to maternal fetal medicine (MFM) subspecialists and/or medical geneticists at tertiary healthcare centres located in urban areas for further diagnostic workup and counselling about management options, in collaboration with other clinical services such as pediatric and family planning subspecialities.

In Canada, there have been no laws restricting abortion since 1988, since which, the overall abortion rate has remained stable at about 14.5 per 1000 females 15–44 years old [2]. However, advances in prenatal diagnosis and increased uptake of these technologies have led to an increase in the rate of terminations for fetal congenital anomalies, from 2.4 per 1000 total births in 2000–2002 to 5.7 per 1000 total births in 2008–2010 in British Columbia [3], one of Canada’s largest provinces by geography and population [4]. Despite no laws restricting abortion, there are persisting inequities in access due to facility and provider-level restrictions, based on factors such as gestational age [2,5]. Furthermore, there is variation in access to abortion training opportunities for health care providers, as well as inequities for patients in geographic distance to services and cost coverage for travel [6–10]. Although national clinical practice guidelines outline technical aspects of procedural (dilation and evacuation) and medication (induction of labour) termination of pregnancy [11], they do not set standards for the full scope of care for termination for medical reasons in the 2^nd^/3^rd^ trimester, including when and how to offer termination, which clinical specialities to involve, and how grief and bereavement should be supported.

We previously described differences in current clinical care pathways for 2^nd^/3^rd^ trimester termination for medical reasons at academic MFM centers in Canada [5]. The wide variation in standard care practices across sites highlights the need for improved understanding of how to promote equitable, high-quality and patient-centred care for 2^nd^/3^rd^ trimester abortion. In this article, we aimed to qualitatively describe provider-identified factors that influence care provision for 2^nd^/3^rd^ trimester termination for medical reasons.

## Methods

### Study design

We conducted an exploratory qualitative study of 2^nd^/3^rd^ trimester abortion providers at all 10 academic MFM sites in Canada. These are tertiary-level healthcare facilities that have a MFM (sub-speciality of obstetrics and gynaecology) training program accredited by the Royal College of Physicians and Surgeons of Canada [12]. We conducted one-on-one semi-structured interviews with clinicians between March 1^st^ and November 30^th^ of 2023, exploring factors that influence care for 2^nd^/3^rd^ trimester abortions for medical reasons, including barriers to and facilitators of this care, at the level of their institution (i.e., not specifically for their individual clinical practice). We employed a realist standpoint by assuming interview data to be accurate representations of reality, to explore underlying mechanisms, i.e., influential factors, in this complex muti-disciplinary healthcare setting [13]. The University of British Columbia Children’s and Women’s Research Ethics Board approved this study (H21-03854).

### Participants

We included three types of participants: MFM subspecialists, medical geneticists/ genetics counsellors, and perinatal nurses/social workers. These professional roles reflect the typical multidisciplinary team of healthcare providers that would provide care to patients undergoing 2^nd^/3^rd^ trimester terminations for medical reasons in a Canadian context. Individuals were eligible to participate if they spoke English or French, and were an independently practicing healthcare provider involved in 2^nd^/3^rd^ trimester termination for medical reasons. We recruited participants by first distributing study details to a senior MFM or medical geneticist in a leadership role at each site via email [14]. We used snowball sampling and asked these clinical leaders to identify one individual from the remaining professional groups at their site, such that we could include one participant from each professional group per site. We asked to be connected to healthcare providers that had in-depth knowledge of abortion care delivery at their site, to understand factors that influence care from various perspectives. The clinical leaders connected us via email, and we shared study details, including the consent form. All participants provided online written informed consent prior to participation and reviewed the consent form with a study team member prior to the interview.

### Data Collection

We conducted semi-structured interviews in English or French. We developed our interview guide with our multidisciplinary bilingual research team. The interview guide was piloted with a representative of each professional group who did not practice at one of the Canadian academic MFM sites. Our guide had two sections that explored 1) clinical pathways for medication and procedural 2^nd^/3^rd^ trimester termination for medical reasons, and 2) perspectives on barriers and facilitators to providing care in this context. We previously reported our findings on the clinical care pathway [5]. This manuscript presents findings related to our second section of interview questions.

ME and ELL conducted all interviews via Zoom video conferencing. Interviews lasted between 45–60 minutes. ME (she/her) was a PhD-trained family planning researcher with experience conducting qualitative research [15–17], and ELL (she/her) was a clinical MFM fellow who was a native French speaker and also fluent in English. Interviewers received in-depth training on qualitative interviewing and thematic analysis from author SM, an experienced qualitative researcher [15–18], prior to starting data collection. Following the interviews, participants completed a short online demographic survey (e.g., age, gender, etc.). We audio-recorded interviews and then professionally transcribed them verbatim. A team member reviewed and de-identified all transcripts.

### Analysis

The two interviewers (ME, ELL) conducted a reflexive thematic analysis [19,20]. This approach centres the idea that researchers are actively interpreting data and acknowledges that knowledge is co-constructed, situated, and contextual. The overall goal of our analysis was to generate useful and actionable evidence to support improvement of 2^nd^/3^rd^ trimester termination care, but we did not have an existing, defined theory of how care ought to be provided. Our analytic approach allowed us to systematically identify patterns in the data, and explore how participants experience, understand, and perceive their local barriers to and facilitators of care.

The two analysts (ME, ELL) independently coded a subset of the same transcripts, compared codes, and agreed on an initial codebook with JL and SM. They then divided and coded the remaining transcripts using NVivo software, Version 14. We subsequently generated initial themes by compiling inductive codes that represent a key idea raised by participants, then refined themes including names and definitions collaboratively as a study team. We analyzed data first by site (i.e., merging perspectives from the clinicians interviewed at each site) and then compared data across sites. We resolved any disagreements through discussion with the study team. Patient partners (CB and EI) contributed to results interpretation and write-up. Team members involved in analysis kept reflexive memos exploring our positionalities, emerging concepts, and patterns in the data that we regularly reviewed to ensure trustworthiness and concordance in data collection and analysis. These verification strategies allowed us to keep a data trail and identify patterns, processes, and key contextual factors. Transcripts were coded in the original language of the interview (English vs. French) and ELL translated quotes from French transcripts into English for this manuscript.

## Results

We interviewed 28 clinicians from across all 10 academic MFM sites in Canada from March to November 2023. This included 10 MFM specialists, 9 medical geneticists or genetic counsellors, and 9 registered nurses or social workers (Table 1). Most participants had worked in their current clinical role for more than 5 years and had at least 5 colleagues at their site who provided the same scope of care for pregnancy termination for medical reasons.

**Table 1:** Participant Demographics from Interviews with Clinicians Providing 2^nd^/3^rd^ trimester abortions for medical reasons in Canada in 2023 (N = 28).

Demographic	n (%)
**Profession** Genetics Maternal Fetal Medicine Subspecialist Nurse / Social Worker	9 (32) 10 (36) 9 (32)
**Age (years)** <40 40-59 >59	* 19 (68) *
**Gender** Men Non-binary Women Not listed	7 (25) 0 21 (75) 0
**Identify as visible minority** Yes No	5 (18) 23 (82)
**Time in current clinical position (years)** <5 5-9 10-19 20+	5 (18) 7 (25) 11 (39) 5 (18)
**Colleagues providing abortion care (n)**^**1**^ <5 ≥5	10 (36) 18 (64)

* Results from age categories <40 years and >59 years suppressed due to small cell counts.

1 Colleagues that provide the same scope of abortion as the participant at their site, within the same professional group

**Fig 1:**
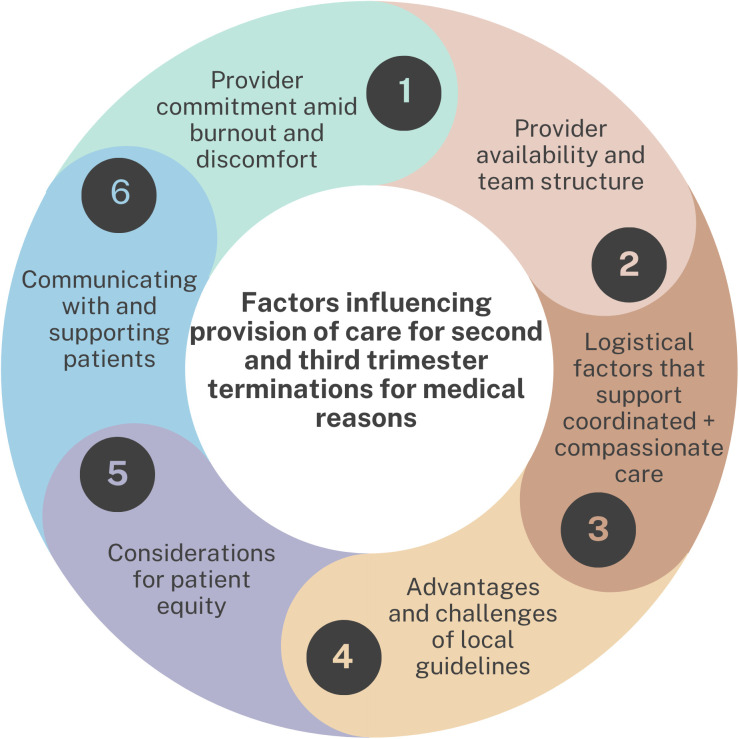
Factors influencing provision of care for second and third trimester terminations of pregnancy for medical reasons, from the perspective of clinicians at Canadian academic Maternal Fetal Medicine sites.

We identified six main themes from clinician perspectives on factors that promote or hinder provision of 2^nd^/ 3^rd^ trimester termination care (see Fig 1 and S1 Table for example quotations): 1) *Provider commitment amid burnout and discomfort;* 2) *Provider availability and team structure*; 3) *Logistical factors that support coordinated and compassionate care;* 4) *Advantages and challenges of local guidelines;* 5) *Considerations for patient equity;* and 6) *Communicating with and supporting patients.* Often participants described both barriers and facilitators within the same theme.

### Theme 1: Provider commitment amid burnout and discomfort

Participants articulated challenges in maintaining their commitment to providing care amid burnout, discomfort, and chronic abortion stigma.

### Provider commitment and burnout

Many participants shared a sense of pride and commitment to providing 2^nd^/3^rd^ trimester abortion care, sharing that they are “pretty darn good at what we do” (ID 27). They recognized that this was one of the most challenging types of care for patients to navigate, and they were strongly motivated to ease the burden on patients and provide a “holistic approach” (ID12). Participants expressed a strong sense of responsibility to ensure care was high quality, readily accessible, and patient centering: “the most important thing is to carry out the woman’s wishes and go with whatever she wants to do” (ID22). Participants shared that this strong sense of responsibility had a positive impact on care provision.

However, many participants also shared stories of the emotional toll of providing this care leading to burnout, outlining concerns about sustainability. They discussed the impact of experiencing chronic abortion stigma: “I was called once the Terminator, and then my life was not easy.” (ID22). Participants also shared that “it always feels like you’re never doing enough” (ID 11) for patients accessing this care, and that these clinical cases are often the most emotionally burdensome for providers: “Generally, I’m able to detach and I’m able to leave and go home and live my life, but this one particular case, I just, I couldn’t let it go.” (ID 19). A few participants expressed concern for inadequate remuneration for the time, personnel, and counselling required to provide this care: “In a fee-for-service model, it’s a lot of work” (ID8).

### The impact of provider discomfort

Some participants expressed personal discomfort with some aspects of the abortion care. One participant said they were moving away from providing procedural terminations as they “don’t find them particularly pleasant things to do” (ID26). Some also said discomfort was more common with the feticidal injection—many clinicians were comfortable overseeing the delivery if the feticide was done offsite or by another provider. However, one participant (ID 17) expressed concern that having feticide provided offsite may increase patients’ experience of stigma. Some highlighted the importance of having a “truly supportive” care team; one participant shared that they told patients: “The nurses who choose to look after patients like you believe in how difficult the situation is for you and that you’re making a very difficult choice, but it is your choice, and are supportive of your decision.” (ID10).

Participants spoke about the impact of variable comfort levels and beliefs about abortion care among team members. Having colleagues that were not willing to provide the full scope of abortion care or who did not agree with offering the option of abortion was seen as a “barrier to always being clear as to what’s available or in which circumstances [an abortion is offered] because that becomes a little bit provider driven.” (ID10). However, some sites did not consider this a barrier, as they noted there were always some clinicians available who were comfortable providing the care requested. One site spoke about requiring everyone to be on the same page: “…everybody in the group had to be comfortable with where we were sitting because they may be on call and have to look after the patient that somebody else has offered termination and willing to carry that out” (ID02). A few participants also reported minimal differences of opinion within their group, and appreciated knowing that everyone on their team was following the same standard of care.

### Theme 2: Provider availability and team structure

Participants identified elements of their team structure that influenced care provision. A shortage of trained healthcare providers willing to offer 2^nd^/ 3^rd^ trimester abortion care contributed to longer wait times for patients and increased provider burnout. While large, multidisciplinary teams were generally seen as beneficial, they could also increase wait times to see subspecialities and inconsistencies in whether the option of termination was offered.

### Desiring more trained providers

Most participants spoke about how a lack of trained providers willing to provide this care meant that the “burden of this work is not shared” (ID02), which contributed to burnout and emotional distress both personally and for their colleagues: “I think the MFMs are stretched. I think the genetics counsellors are stretched. I definitely know social work is stretched.” (ID11). In large part, people described the need for more providers within their own discipline, but some also included the need for improved access to specialists in pathology, palliative care, and other subspecialties. These concerns were emphasized for later terminations (e.g., after 20 or 24 weeks’ gestation), including concerns around finding a provider willing to provide a feticidal injection. One participant shared that they had to tell their patient: “‘Now you have to wait until there’s a provider who will do your KCl [feticidal medication] injection.’ It was a long weekend, so she had to wait. It was just a very long and drawn-out process.” (ID16). Some participants shared that a lack of trained procedural abortion providers contributed to longer wait times for patients. Participants also provided insights into the reasons for the lack of trained providers, including insufficient abortion training during core residency in obstetrics and gynaecology (“I don’t think we get a lot of specific training” -ID21), as well as many clinicians being unwilling to provide abortion care due to discomfort or antichoice attitudes (“There are some that say, ‘I won’t do that.’” -ID19).

### Large multi-disciplinary teams can help or hinder care

Most participants described that “It’s ideal to have a multidisciplinary approach,” (ID18), involving collaboration from diverse clinical specialities (e.g., pediatric subspecialities, pathologists) and allied health professionals (e.g., social workers), particularly during the termination decision-making process. Multidisciplinary approaches limited the number of appointments, travel, and repetitive conversations patients were required to have. However, participants also discussed that multidisciplinary care required effective communication, trust and “cohesion” between providers (ID13), and “a sense of mutual respect amongst the team” (ID16). Many participants cited the importance of developing personal relationships with fellow providers and using informal communication approaches such as texting with colleagues. In addition to facilitating an effective multidisciplinary approach this also led to learning from fellow providers and finding mentors to discuss cases with.

Participants also described challenges to implementing a multidisciplinary approach, including limited availability (e.g., nursing and social work) or long wait times for patients to see certain providers (e.g., pediatric subspecialists). One participant said of palliative care: “capacity just doesn’t allow. I mean, we’d love to have them involved in all these cases, but it’s just not possible.” (ID13). Participants shared that, at times, long wait times meant that patients were rushed to make decisions about whether to terminate a pregnancy before having the opportunity to consult with various subspecialists due to gestational age limits for procedural abortion or patients wanting to decide quickly.

### Theme 3: Logistical factors that support coordinated and compassionate care

Paperwork requirements, the availability of a care coordinator, and the physical environment in which terminations are provided were found to be instrumental barriers to or facilitators of care.

### Care coordination

Participants shared that the paperwork and care coordination required to provide 2^nd^/3^rd^ trimester abortions was more burdensome to providers than other types of obstetric care. Having specific care coordinators, such as a nurse clinical care coordinator or genetic counsellor, was a key facilitator to high-quality care, providing a point person for patient communication and helping to review options and facilitate referrals as needed: “we have a single point of contact …They can call just directly to the phone number for the patient care coordinators. Then we keep notes between. There are only two or three nurses that do that role…so that we don’t have to have people repeating their story each time.” (ID24). One participant (ID 21) described the benefits of having a checklist for medication abortion which helped to ensure providers completed all required paperwork and care steps. Participants also discussed aspects of care that placed additional strain on care coordination, such as having not having mifepristone, a key medication used in both medication and procedural pregnancy termination, stocked in hospital formularies: “it’s not that easy for us to get our hands on [mifepristone]” – ID26.

### Situating care away from labour and delivery

About half of the sites reported having dedicated private rooms for terminations away from the labour and delivery unit, which was often perceived as beneficial to patient care. Many participants shared concerns about caring for patients on labour and delivery units, where they are in close proximity to other patients with live births, and thus seeing and hearing the delivery of healthy babies: “We’re sensitive in trying to keep them in a quiet corner” (ID20) and “we really try our best to keep them away from hearing things they don’t want to hear” (ID27). Participants that did not have access to dedicated rooms for terminations away from other birthers thought this would increase patient comfort if available: “It’d be really optimal, in my opinion, if we had a space that was completely separate” (ID11). Conversely, one participant felt having these patients on labour and delivery facilitated good care: “The reason why I think the induction-of-labour patients are getting excellent care is that they get the equivalent care as to patients who go there to deliver their newborn with an expected outcome of a live birth in terms of the amount of support, nursing care, facility, the one room for they and their partner.” (ID10).

### Theme 4: Advantages and challenges of local guidelines

Participants reflected on the advantages and challenges of local guidelines (or lack thereof). Most centres reported not having written guidelines or protocols available for 2^nd^/3^rd^ trimester termination of pregnancy for medical reasons, or said they were outdated. They discussed how local guidelines could shape equity in access and outcomes for patients, but also how they could limit the extent to which care could be tailored and truly patient-centered.

### The lack of guidelines can increase flexibility

Some participants felt that not having guidelines was beneficial because they could be flexible to meet patient needs: “there has been a lot of resistance from the MFM team to have it prescriptive because we like the flexibility of not having specified gestational ages… it reduces barriers to [patients] getting the services that they are requesting” (ID04).

Several sites rescinded guidelines or adjusted them to make termination care more available (i.e., regardless of gestational age or indication) during the COVID-19 pandemic due to travel restrictions: “during COVID, they made exceptions for more people” (ID24). With the end of the pandemic, participants were hesitant to return to more restrictive guidelines as current care practices (in which more services could be provided in their hospital) improved access to patient-centred care.

### Having guidelines can facilitate consistent care

Some participants shared concerns on equitable care provision when no guidelines or protocols were present: “from one patient to the next, it may look very different” (ID13). For example, there could be inconsistency in abortion options counselling depending on the provider doing the counselling, and this “breakdown in communication” (ID12) was seen as a serious detriment to patients’ quality-of-care. A few sites were in the process of trying to create an internal protocol to offer “broad structure” (ID26) to providers, but also found it “very hard to standardize it” (ID19). Multiple participants shared that protocols “have to be flexible enough to account for all eventualities” (ID26) but that having care checklists in place “prevent anyone from falling through the cracks” (ID06).

### Theme 5: Considerations for patient equity

Participants described that adjusting care or being sensitive to lived experiences and demographics of their patients was a facilitator to care. This included adjusting care for patients who lived in rural and remote locations, or those with different cultures or faiths. Participants also described challenges to equitable healthcare, such as inadequate access to language interpretation and financial burdens placed on patients.

### Considerations for patients living in rural and remote locations

Participants perceived that patients living in rural and remote locations faced additional challenges accessing care. To mitigate this, most sites described “a courtesy that’s extended to people from out of town” (ID12) where they adapt care to try and “minimize the number of trips they have to do” (ID10) and using telemedicine when available. Not all sites were able to do this, especially when patients had to see different specialities and these specialities required separate referrals. Telemedicine was viewed as a facilitator to accessible care, especially for post-abortion follow-up and social work visits: “[telephone and virtual care] made it actually a lot easier to provide service to a lot of our out-of-town patients. We can provide some of those additional supports because it can be just a phone call, right?” (ID13). Some participants described working with providers in rural hospitals to help patients receive termination services closer to their home: “The obstetricians here work with the obstetricians at some of the rural centres to help organise an induction of labour” (ID13).

### The financial burden faced by patients

Participants also expressed concern that “there is still a financial burden” for patients accessing this type of care despite Canada’s universal healthcare coverage, if patients had to travel from out of town or take unpaid time off work for appointments. Additionally, participants described the financial burden to patients of paying for the funeral, burial/cremation, or for private counselling: “our governments don’t do enough to fund that kind of support, so it’s very expensive, and it’s cost prohibitive for many of our patients” (ID16). Costs and coverage for these aspects of termination care differed by province and institution. Most participants highlighted the important role of social workers in helping patients navigate costly parts of the process. At some sites social workers could access funding for patients travelling for care or requiring childcare.

### Difficulty finding adequate language interpretation

Some participants described challenges finding adequate language interpretation for patients, which inhibited equitable care: “We do have a lot of newcomers in [city], and many of our patients do not speak English and don’t understand our processes, so that can be a significant barrier, particularly if it’s a language that’s very difficult to access interpretation for. That’s very, very difficult.” (ID16). Additionally, some participants expressed concerns that the interpreters were not presenting an unbiased opinion: “We bring an interpreter. That comes with challenges because you want to make sure they’re unbiased. Not all interpreters are comfortable with that discussion, especially when it comes to options in an interruption of pregnancy. Some may say like, “I’m not comfortable translating that.” (ID17). Some participants highlighted the success of having an interpretation machine onsite.

### Supporting patients’ cultures and beliefs

Participants discussed ways they “accommodated” (ID11) different cultures, faiths, or religions. A few sites had spiritual guidance available onsite as an option. Multiple sites described having access to Indigenous patient navigators or facilitating practices such as smudging, and perceived that this increased access to culturally safe care. One participant described making exceptions to fetal remains disposition protocols: “I know sometimes some of our Indigenous folks would like to have traditional burials, so we have, in the past, for losses under 20 weeks, facilitated them to pick up the remains themselves, not using a funeral home” (ID20).

### Gender identity

When prompted about gender diversity during the interview, many participants were confused, unable to answer the question, or did not have experience working with gender diverse patients: “I can’t really speak to that one” (ID24). Two participants described that pronouns are noted in medical records at their centre (ID23 & 27), including that “even for the baby, if the patients refer to themselves as they, and they want the baby referred to as they, we can accommodate that now” (ID27).

Some participants shared that care was not modified based on patients’ age, gender, cultural or ethnic background, or other demographic characteristics, and that this standardisation was a facilitator to good care. One participant described relying on their social work colleagues to navigate that portion of patient care (ID05).

### Theme 6: Communicating with and supporting patients

Participants highlighted the importance of communication, supporting grief and bereavement, and follow-up as key factors that impacted care.

### Communication

Participants noted the importance of clear and accessible communication, often assisted by a clinical coordinator, to facilitate care. Participants sometimes used more informal methods of communication with their patients to support decision making: “I find a lot of patients seem to feel very comfortable texting you things and questions that they may not feel comfortable asking you in person” (ID11).

Participants shared the importance of mirroring patients’ terminology around their abortion (e.g., using the term abortion versus termination versus pregnancy interruption). Participants also highlighted the importance of addressing potential stigma patients may face by discussing how their experiences could be framed with family, friendships, or workplace, and how to prepare for abortion protesters that may be present at the hospital.

### Bereavement and supporting family

Participants described value in offering patients mementos and memory-making opportunities, including: a small box with foot and/or handprints, locks of hair, clothes the baby wore, seeds for planting, professional photographs, spiritual blessing certificates, heart-shaped stones, plaques with the chosen name, and other items. A participant described: “we have little flannel hearts. We weigh the baby, and then we fill the flannel heart with sand that is the exact same weight as the baby. It’s really sweet” (ID 11). For patients that may not know if it is something they want, some participants described keeping memento boxes for months to years after the termination in case a patient wants it later. Having volunteers or community organisations involved in the creation of these memory-making experiences was often described as essential. Some participants said options for memory-making were more limited for procedural abortions, but efforts were still made to offer patients a keepsake.

Participants discussed how they supported patients’ families, including for non-birthing parents/partners, children, and extended family, to facilitate care. Some sites had resources specifically for family members: “We have books for moms, books for dads, books for couples together, books for grandparents. We have books for children. We have access to an awesome child-life specialist who works in the NICU with us if we need to support siblings” (ID11). A few participants raised concerns that family members may not always have the support they require to understand what has happened and navigate their grief and bereavement: “I think partners are kind of left in the dust a little bit… I worked with a family a few months ago and I recall the partner just feeling very isolated” (ID20). To combat this, some providers spoke about doing “little things like bringing food for the partner when you see that he hasn’t brought anything to eat” (ID11).

### Lacking post-termination support systems

Participants identified a need for improved post-abortion follow-up from healthcare providers as “the ongoing support system is significantly lacking”. At most sites, once patients were discharged from their hospital, post-abortion follow up care was limited to a single appointment in 6 weeks. One participant described that patients are “completely lost to follow up” in the longer-term, and there “is no support for these people, and there is no ongoing care.” (ID 19). The responsibility often falls on the patient to coordinate funeral, burial, and cremation arrangements, sometimes with assistance from nursing or social work. This can be financially costly, traumatic, and time consuming. Wait times for fetal autopsy results, which require MFM or medical genetics follow-up, are often perceived as distressing to patients. One of the biggest concerns amongst participants was the need for ongoing grief and bereavement support. Patients often relied on community support groups or private counselling, which could be expensive, only offered in certain languages, or too generalised (e.g., not specific to termination, but aimed at those experiencing perinatal loss more generally).

### Participant Reflections

Few participants felt that there were no barriers that needed to be addressed: “I think that after so many years that we struggled with the process of pregnancy termination, we finally, even in the last 15 years, we’re able to do what needs to be done. It wasn’t easy” (ID22). Another participant shared that “I think, for me, the nugget here is that deeply personal experience and how people want to move through it and how they wanna remember it and how we can support them, so that when they look back, it’ll be like, ‘Yeah, that was horrible but, man, I felt really well supported.’ I think that’s all we can ask and hope for, you know?” (ID11). Participants were keen to hear about results of this study, wanting concrete ideas on how they can continue to improve care.

## Discussion

### Main Findings

This national qualitative exploratory study investigated the factors influencing the provision of 2^nd^/3^rd^ trimester termination of pregnancy for medical reasons at academic MFM sites across Canada. Our thematic analysis revealed six key themes highlighting both barriers to address and facilitators of providing care in this context. Our findings underscore the need to increase the number of 2^nd^/3^rd^ trimester abortion providers, build cohesive multidisciplinary teams, and support comprehensive care coordination, strong provider-patient relationships with open communication, and more post-termination follow-up. There is also a need to mitigate the burden of patient travel and gaps in language translation services. Our results emphasized the need for guidelines and protocols that promote health equity and remain flexible enough to meet patient needs regardless of gestational age and clinical indication.

### Interpretation

Our study is the first systematic national investigation, to our knowledge, of healthcare provider perspectives on factors influencing care for termination for medical reasons in Canada. A 2022 systematic review on patient and partner healthcare experiences or needs regarding 2^nd^/3^rd^ trimester abortion for fetal anomalies included 30 articles [21], of which only two were conducted in Canada—one interviewed 19 women regarding the conceptual framing of fetal remains [22], and other interviewed three women from Canada and 7 from the United States about their experiences of termination for medical reasons [23]. These studies highlight the importance of creating space for the multiplicity of patient experiences and wishes regarding termination for medical reasons. The healthcare provider perspectives in our study supports and builds on these previous findings. Our findings are also consistent with other themes noted in the review overall [21], such as the need for coordinated care and more post-termination follow-up care; we further describe concrete ideas of how these needs can be realized (e.g., dedicated care coordinators and collaboration with community organisations for follow-up support).

We also highlight the importance of clinical guidelines that offer structure to promote equitable access to abortion care, while being flexible enough to ensure care is patient-centred. This tension, by which guidelines/regulations can both promote and restrict access to abortion care, is not new: in 1969, prior to decriminalization, Canadian abortion laws were liberalised to allow for provision of abortion, but they also mandated approval by a hospital “Therapeutic Abortion Committee”, which meant that care continued to be inequitable. More contemporarily, and as highlighted by our participants, hospital guidelines restricting access to termination based on gestational age or indication, or regulating the disposition of products of conception, can also impact equity and cultural safety [24]. A similar tension has been described between individualised vs. standardised care across medicine more broadly [25–27], acknowledging that rigid standardisation of care that is not responsive to patient differences centres a paternalistic as opposed to an equitable approach to care provision. One group proposes individualised standardisation as a solution, defined as “the imposition of standards, regulations, or norms which are tailored to the genes, body condition, culture, social environment, values, needs, and preferences of the individual patient” [27,28]. This leaves us with the question: How can clinical guidelines for termination care be redesigned to provide clinicians with clear, evidence-based direction while preserving their ability to deliver individualised, patient-centered care?

When asked about barriers to and facilitators of 2^nd^/3^rd^ trimester termination for medical reasons, clinicians in our study did not focus on technical aspects of care, but rather on details such as team dynamics, communication with patients, and grief and bereavement support. Broadening the scope of clinical practice guidelines to include topics such as these may help facilitate equitable care. These updated guidelines could draw upon international guidelines on grief and bereavement for perinatal loss, which suggest strategies such as in-hospital multidisciplinary bereavement teams [29,30].

Finally, the concerns regarding provider availability identified in our study align with a 2023 scoping review on later gestation abortion in Canada, which identified a lack of training to provide 2^nd^/3^rd^ trimester abortions, and limitations of providers decreasingly offering termination services as gestation increases [7].

### Strengths and Limitations

Our project provided an in-depth exploration of factors influencing 2^nd^ and 3^rd^ trimester abortion care at all ten academic MFM sites in Canada. We interviewed ~3 clinicians per site, capturing the perspectives of multiple clinical specialities, to illustrate a fulsome picture of care delivery. However, our findings may not represent all experiences and hospital sub-sites, and does not reflect practices outside these hospitals, including those in the Territories, Saskatchewan, New-Brunswick, and Prince Edward Island. Although we initially aimed to recruit 30 participants, for feasibility reasons, we closed recruitment at 28 participants. We were unable to interview a registered nurse or social worker from one site, and a geneticist from another, despite multiple attempts over 8 months. We obtained adequate data from MFM and Medical Genetics to answer our research questions but did not have adequate data to have confidence in representing perspectives from nursing and social work disciplines. Participants were also not able to fully describe the procedural termination pathway, which are usually provided by general obstetrician-gynaecologists rather than MFM specialists and often not performed on-site. Gaining the perspectives from general obstetrician-gynaecologists in future projects would give us a more fulsome understanding of care delivery. Although this project yielded in-depth insights into the factors that influence pregnancy termination care from providers’ perspectives, we recognise that exploring and integrating patients’ perspectives will be critical to effectively improving care.

## Future Directions

Our research exemplifies the need for national conversations on improving the quality of abortion care, extending beyond the first trimester of pregnancy and technical aspects of the procedures. Community and advocacy organisations that support access to abortion, sexual health, and perinatal bereavement services have long been leading these initiatives, and academic and clinical voices can contribute to these important efforts. Our research provides specific barriers to and facilitators of care for clinicians and policy makers to consider, which can also stimulate further discussion toward improving and destigmatising abortion care.

## Conclusion

Our exploratory qualitative study with clinicians at Canadian academic MFM centres identified several barriers to and facilitators of care for second and third trimester terminations for medical reasons. Our results highlight the need for guidelines that promote health equity and allow for flexibility to accommodate patient needs regardless of gestational age and indication. Considering these factors may help individual institutions identify opportunities to improve provision of abortion care.

## Supporting information

S1 Table Themes and example quotes from interviews with clinicians providing 2^nd^/3^rd^ trimester pregnancy termination care for medical reasons in Canada in 2023. (PDF)
