# Supplementary material for: Factors that influence care for second and third trimester termination of pregnancy for medical reasons in Canada: A qualitative investigation
Source: PLoS One. 2026 May 15;21(5):e0349167. doi: 10.1371/journal.pone.0349167 (PMC13178905; doi:10.1371/journal.pone.0349167)
Supplement: S1 Table — (PDF) [file pone.0349167.s001.pdf]

Supplemental Table 1: Themes and example quotes from interviews with clinicians providing 2nd/3rd trimester pregnancy termination care for medical reasons in Canada in 2023

Theme	Sub-theme	Example Quotes
Provider commitment, burnout, and discomfort	/	Some people really understand that these are exceptional services that we're trying to provide. Other people, I'm sure, just think, "Oh, that's what hospitals do. All hospitals do that. That's just normal." Of course, we know that that isn't. I'm not saying this hospital is perfect. I think there's a lot of ways we could be better, but I think everybody is really super on board with trying to really support people for medical terminations. – ID12<br><br>There are a couple of scenarios where I have ended up doing the feticides on patients who were going down this pathway for situations that I felt somewhat uncomfortable about doing feticide for because the prognosis wasn't as bad as perceived, but it's kind of a difficult balance – ID26<br><br>So I can speak to the MFM group. There are some that say, "I won't do that." That's fine. In terms of the nursing group as well, there are some that say, "No, I won't do that." … They're not saying it in a, I don't perceive it to be in a way where they're judging myself or my colleague for doing the procedure. They just feel like they can't emotionally provide the procedure but are grateful that other people are able to provide the procedure. – ID19
Provider availability and team structure	Desiring more trained providers	M: What can be done differently to improve access at your site?<br><br>R: More staff. I think the MFMs are stretched. I think the genetics counsellors are stretched. I definitely know social work is

		stretched. I think we're all running short continuously. It's that old being short makes you less resilient, and then there's more sick time and that whole cycle. – ID 11<br><br>I think the challenge sometimes can be organizing a team in a timely fashion so that it's less distressing on the patient. Again, this is purely based on resources. I don't think it's a system's issue. I think it's a resource issue. – ID19<br><br>if we're suspecting a genetic issue, we always send them to a geneticist, a genetic team. We often will send them to the [site]. If it's a surgical issue, then we send them to a surgical team for those. The issue with some of these external referrals, again, because it's not within our system, the wait time is based on their system. They make every effort to accommodate our patients, but sometimes I feel that, because of the delay, the patients are hesitant to wait and would just like to proceed with terminating, which is unfortunate because I think that those consultations bring value and obviously more insight to whatever the issue is we're discussing. – ID19
/	Large multi-disciplinary approaches can help or hinder	I feel to offer excellent care or what you mentioned, having all the extra support is ideal, so to make the diagnosis, like genetic, cardiology, (inaudible), paediatrician, all that so that the patient can be informed from different perspective. The multidisciplinary approach is also important for the support, so I think having access to, to help them through, the support. I mean, our social worker is great for also just the funeral arrangement, all these extra steps that I, myself, as a physician, might not know all the details of the process of that, but she'll walk them through it. There's also a funeral home that doesn't charge them for when it's a termination, so we have that direct contact

		with that group. Yeah, it's from the diagnosis, from the counselling, but also from the follow-up. I think it's ideal to have a multidisciplinary approach. – ID18<br><br>I would say definitely our multidisciplinary approach [is a key facilitator] with the weekly meetings because, yes, we really have a good exchange between the different specialties or specialists. We're not just depending on reading somebody else's written report. We can also get sometimes more information that might not be in the chart, like on the social situation and sort of personal impressions that the physicians get in their care, in their interactions, so I think that's really helpful, if you have this. And I know, I've been in this program for a couple of years now, I know the paediatric cardiologist, I know the NICU staff who do those consultations, I know the MFM, so after a while, I know the paed surgeons. We know each other, so people know who to go to if there's a question or a concern or a problem or you need rapid access to something. It's a good network, I think, and that relation should be helpful. – ID25<br><br>We have an increasing number of the paediatric subspecialties who are willing to see people prenatally. We've always had a very good relationship clearly with genetics and paediatric surgery, but morphology, urology, neurosurgery has also been very willing and able to see people prenatally to counsel them on quality-of-life impact, etc. That's extended a lot more over the last few years.– ID26
Logistical factors that support coordinated and compassionate care	Care coordination	I think it's helpful, too, that we have our FDTG nurse coordinator, because part of her salary is really for this purpose, to coordinate the care and make sure that the consultations are filed, that the patients are actually seen, and that everybody knows when there's

		somebody complicated, with a complicated story coming in. I think, I would say that's also very helpful. For now, it's not a permanent position, so I hope that the hospital finds it in its budget somewhere to make this a permanent post because it's a really important. – ID26
/	Situating care away from labour and delivery	We would do an induction of labour, and it would take place on one of two locations. It would either take place on a ward called [unit], which is an all-female ward where women are either seen for complications in pregnancy. They've delivered and have their baby on the neonatal unit. There are no babies on the ward. Or they're coming in for a termination or depending on how far along she is and if she's had a previous C-section and there are any potential complications, we also do the deliveries right on Labour and Delivery, which is sometimes a little bit tricky because you can hear women in labour in other rooms, but they are all private rooms… So it'd be really optimal, in my opinion, if we had a space like that that was completely separate. I know it's not gonna happen logistically. – ID 11<br><br>Those cases, they have a dedicated room space in our birthing centre. We mark it with a butterfly mark on it that this is an interruption, pregnancy interruption and going through labour… We have a quiet room dedicated for examining the baby after. Those are additional accommodations we do down there. I think it's a rule, like, most of the nursing there, we don't have clearly visibly pregnant nursing that are going through such scenarios. Those are some accommodations I can think of. – ID17
Considerations for patient equity	Considerations for patients living in rural and remote locations	For some patients who are in sort of rural areas, there have been a little bit more accessibility to care where we would still coordinate it. The obstetricians here work with the obstetricians at some of the rural centres to help organize an induction of

		labour for that, say, terminal diagnosis. This patient shouldn't have to come to [city] and be away from their family and friends to meet and say good-bye to their baby with trisomy 18. They should be able to do this locally if other births are happening locally. – ID13<br><br>We unfortunately cannot bunch clinics or bunch appointments. The only appointments that we can cluster or try and, I mean, get on the same day is us, like an MFM and a perinatology visit, so we do do that, but because we also have to outsource for our geneticist, for our cardiologist, they're not in-house, so this is the issue with trying to get them on the same visit. – ID19
/	Difficulty finding adequate language interpretation	Also, we have a lot of immigrant families here, and we have frequent use of interpreters. I find that we're always able to get an interpreter for an appointment. I do think that MFM is not always as prepared with an interpreter at the time of their appointments. I've had some circumstances or some situations where I don't, I'm not sure that the patient fully understood how they were counselled in MFM because they didn't have an interpreter. – ID23
/	The financial burden faced by patients	Yeah, I think that the, I mean, I'm not, I think there are barriers just from the point of view of, um, there's a lot of support to provide them lists of cremation services and stuff, but there is still a financial burden attached to that. I think for some couples who are terminating, you know, in the 2nd trimester, it takes them by surprise. – ID10
/	Supporting patients' cultures, and beliefs	Our Muslim population, they have, um, they like the baby dressed in white, and they like to take the baby. They do a, they want the baby, I think it's buried before (sundown). They like to have the baby soon after delivery, so we try to get the baby ready and do the hand and footprints, if they wish for that done, and pictures done quickly and then

		dress the baby in white. We have white boxes now that we keep on site to wrap the baby up for them to take home or to their local church or wherever they wish to do their memorial afterwards. Yeah. – ID21<br><br>if they wanna see a spiritual care, if they wanna see spiritual care from our hospital, but maybe they want somebody from outside. Maybe they want a Catholic priest, or they want somebody else. Then we definitely facilitate that, and we'll bring in somebody from the outside or invite them to bring whoever they want in. – ID12<br><br>Smudging is a common request here with our Indigenous population, and they do make every effort to allow them to do that. – ID19<br><br>Ancillary point of view, for instance, if we know and identify a person is First Nations, we always offer, um, there's First Nations liaison and support people on the site, so we offer that to them. – ID02
/	Gender Identity	In terms of gender, I mean, that, we have a bunch of same-sex couples, for sure. If you're asking if I've looked after a mother who's identified as anything other than female, fortunately not yet because I'm not sure of that one. I'm sure we'd manage that, but no, I haven't encountered that yet. – ID26<br><br>generally, we have social work involved, which is very helpful in kind of traversing those kinds of issues that come up, but I don't know [how we tailor care to diverse populations]. – ID05
Advantages and challenges of local guidelines	/	I mean, I think one of our biggest challenges is that we have a limited number of providers who are providing these services, and there are really no standard protocols or guidelines, so from one patient to the next, it may look very different. It's hard as a

		provider on our end who's providing the counselling and trying to explain to a patient what this termination looks like and they're asking you, "Okay. When will this happen? Who will I see? Where am I gonna go?" and it's not always easy to answer, so it can be, "Well, we usually this and this and this is the case, but I need to check with so and so. Myself or they will get back to you." There's not always a standard either in terms of who contacts the patient or gives them some of that information. It can be a bit hiccupy at times, yeah. – ID13<br><br>I think our guideline is a little bit of a barrier sometimes because it does force us to kind of limit how much we can offer particularly a little bit later in pregnancy. – ID 6
Communicating with and supporting patients	Communication	Even when I'm doing letters for families, because a lot of families need letters for work or different things, I always just talk about, unfortunately, they're having pregnancy complications and are likely to experience a loss. I figure you tell your story to who you trust, right? – ID11<br><br>Yeah. Yeah, so we have interpretive services. We don't always have access to an in-person interpreter, but we almost always, I think there's been very few languages that we couldn't connect with a phone interpreter, so we have phone interpretation available. ID13<br><br>We always call the patients after the procedure and ask them if we could improve our procedure and if there are any problems that we can avoid, so the feedback is immediate with any patient we see. We just sit on the phone every weekend and call all the patients that we had termination to ask them how they are doing and if we can be of further help. – ID22
/	Bereavement and supporting family	Another thing that we haven't talked about is mementos and the kind of care that we

		provide to families so that they can create a lifetime of mementos in this little, tiny window that they have from getting pregnant to the end of life and giving them access to professional photography or taking photographs for them or providing them with footprints or foot molds or little crib cards with their baby's name on it and little heart lockets and little butterfly seeds and candles to take home to remember their baby when they light a candle and take a moment of reflection out of the day… we have a slew of volunteers in the community who also support this program, and the volunteers do things like they knit little, tiny tuques for 20-week babies with their little tiny little heads. They knit little blankets to wrap them in, and they make little flannel blankets. They do this to support families, or families who've had a loss themselves will, you know, I had a woman who came back recently who made three dozen blankets or more because she had received one. She was so moved by getting it that she wanted to make some for other people that were going through this, or people that make little memory boxes to give to other families because they were so, you know, sometimes people buy little outfits or something because they wanna pass something on to another family. – ID12<br><br>On the D&E side, we offer, above 15-, 16-weeks' gestation, we can offer that the physician can try to provide footprints on a little card, so we have little cards that we have. They have a little label on the inside that says something very sentimental about small footprints. I can't remember what it says. The physician will try to put footprints on that card for patients. We do tell patients that it's if possible because sometimes the nature of the procedure or the nature of the fetal anomaly doesn't allow that, but we try, so we offer that. – ID 16<br><br>We have a resource specialist through, she's an information specialist through our site

		library. When somebody calls and says, “Well, my four year old is still not getting over the loss, and my four year old wants to go to heaven to be with his baby sister who died. I need some resources.” Her name is [resource specialist]. She’s amazing. I’ve worked with her. She’s ordered all of this great, great literature to support siblings through loss and connects with families and will send them books, not just give them titles, “Here, go out and find this,” but she will send them the books that they need… Some people will bring, you know, they have a three year old and a five year old at home or whatever, and they bring the kids in to meet the family, to meet the baby. They have this time together, and they have photos together. The children are very much a part of the loss, and they get to hold the baby and ask questions and see the baby. – ID12
/	Lacking post-termination support systems	the challenge and barrier and even from my perspective—this is just observationally—is that after termination, the supports, the ongoing support system is significantly lacking. I have had these patients articulate to me that they feel uncomfortable going to, for example, a support group whereby somebody has had or most of the patients have had, for example, a stillbirth, so it was not an induced termination. I think that there’s a lot of stigma surrounding that, ongoing stigma, and even how to communicate that to family. It’s actually very interesting because, in certain cultures, termination is simply just not an option, whether it’s a lethal anomaly or not, so I think that support, there is no support for these people, and there is no ongoing care for them. Unfortunately, because we are a high-risk obstetrical clinic and I don’t follow these people long-term, they are completely lost to follow-up. Some don’t have primary care providers, so I feel like they’re just left to their own devices out in the community and the resources stop. – ID19

		I think most programs are offered in English, so if you speak a different language, it's very tricky to get support. I think if you want private counselling and you don't have an employee assistance program or money, you're out of luck because someone like me in the community charges a low $90 an hour to a high of $200 an hour, right? So I think all the groups are accessible to everybody in terms of financially. I don't know that they're accessible in terms of languages and comprehension, but I know that individual counselling really is targeted at if you have either coverage through insurance. – ID 11
